# Reallocating desk workers’ sitting time to standing or stepping: associations with work performance

**DOI:** 10.1093/occmed/kqad142

**Published:** 2023-12-16

**Authors:** C-Y Lin, A Shibata, K Ishii, M J Koohsari, N Hadgraft, D W Dunstan, N Owen, K Oka

**Affiliations:** Department of Public Health, College of Public Health, China Medical University, Taichung, 406040Taiwan; Faculty of Sport Sciences, Waseda University, Tokorozawa, 359-1192Japan; Centre for Urban Transitions, Swinburne University of Technology, Melbourne, Victoria, 3122Australia; Institute of Health and Sport Sciences, University of Tsukuba, Tsukuba, 305-8577Japan; Faculty of Sport Sciences, Waseda University, Tokorozawa, 359-1192Japan; Faculty of Sport Sciences, Waseda University, Tokorozawa, 359-1192Japan; School of Knowledge Science, Japan Advanced Institute of Science and Technology, Nomi, 923-1292Japan; Institute for Physical Activity and Nutrition, School of Exercise and Nutrition Sciences, Deakin University, Geelong, Victoria, 3216Australia; Centre for Urban Transitions, Swinburne University of Technology, Melbourne, Victoria, 3122Australia; Physical Activity Laboratory, Baker Heart & Diabetes Institute, Melbourne, Victoria, 3004Australia; Institute for Physical Activity and Nutrition, School of Exercise and Nutrition Sciences, Deakin University, Geelong, Victoria, 3216Australia; Physical Activity Laboratory, Baker Heart & Diabetes Institute, Melbourne, Victoria, 3004Australia; Centre for Urban Transitions, Swinburne University of Technology, Melbourne, Victoria, 3122Australia; Physical Activity Laboratory, Baker Heart & Diabetes Institute, Melbourne, Victoria, 3004Australia; Faculty of Sport Sciences, Waseda University, Tokorozawa, 359-1192Japan

## Abstract

**Background:**

Studies have suggested that sitting time at work may lead to underperformance but they may underestimate the benefits to desk workers’ performance of reducing occupational sitting time without considering the relative effects of the specific activities replaced.

**Aims:**

To estimate differences in work performance (presenteeism, absenteeism and engagement) when occupational sitting time is reallocated to standing/stepping in desk workers.

**Methods:**

Data for middle-aged desk workers were from a Japan-wide online survey (*n* = 2228). Self-report proportion of occupational sitting and standing/stepping, work hours and work performance indicators, including absolute (ratings relating only to self) and relative (ratings of self, compared to others) presenteeism and absenteeism, and dimensions of work engagement, were collected. Partition and isotemporal substitution models were used to investigate the associations of occupational sitting and standing/stepping time with work performance, including their reallocation effects.

**Results:**

In partition models, longer occupational sitting time was associated with a lower absolute presenteeism score (i.e. less productivity), lower absolute absenteeism (i.e. longer-than-expected work hours), and lower engagement. Longer occupational standing/stepping time was associated with lower absolute absenteeism and more engagement. Isotemporal substitution models showed that each hour of occupational sitting reallocated to standing/stepping was favourably associated with overall work engagement (*B* = 0.087; 95% confidence interval 0.051, 0.122) and its dimensions (*B* ranged from 0.078 to 0.092), but was not associated with presenteeism or absenteeism.

**Conclusions:**

These findings suggest that management support and practical initiatives to encourage desk workers to replace portions of their sitting time with standing/stepping may contribute to enhanced work engagement.

## Teaser

This study estimated differences in work performance (presenteeism, absenteeism and engagement) when occupational sitting time is reallocated to standing/stepping in desk workers. We found that time of occupational sitting reallocated to standing/stepping was favourably associated with overall and dimensions of work engagement but not presenteeism or absenteeism. These findings suggest that management support and practical initiatives to encourage desk workers to replace their sitting time with standing/stepping may contribute to enhanced work engagement.

**Key learning points**

**What is already known about this subject:**

- Possible associations between less occupational sitting time and higher levels of work efficiency and work engagement were found in some previous studies.
- These relevant cross-sectional studies did not take into account the time-use composition of these behaviours.

**What this study adds:**

- There are putative benefits of reallocating occupational sitting to standing/stepping on work engagement.
- Limited associations with sitting–standing/stepping reallocation were found for presenteeism or absenteeism.

**What impact this may have on practice or policy:**

- Work practices and strategies that support time being spent standing and stepping at work, with decreased sitting behaviours, have the potential to improve desk workers’ engagement.

## Introduction

Individual work performance can directly affect the productivity of the workplace across all sectors. Indicators such as presenteeism (i.e. being at work without being productive) and absenteeism (i.e. not reporting for work as scheduled) can be associated with productivity loss [1], while work engagement (i.e. high morale with dedication and a strong focus on work) has been linked to higher work productivity [2]. Underperforming workers who have any presence of high presenteeism, high absenteeism or low engagement may reduce a workplace’s overall productivity in both quantity and quality. Flexible work hours and great self-control over working patterns for workers are suggested to increase work performance but are not put into widespread practice in the workplace [3]. Strategies of behavioural changes that took place at work, with non-reduced work hours, associated with work performance may be more feasible and acceptable for the workplace to develop and carry out relevant initiatives for improving worker morale and productivity.

Reduction in time spent for sedentary behaviours—any waking behaviour with an energy expenditure of ≤1.5 metabolic equivalents while in a sitting or reclining posture [4]—is a main target on improving desk workers’ health, well-being and productivity. For example, a study from Northern Ireland found that less time in occupational sitting was associated with a higher level of vigour—an aspect of work engagement [5]. Another study from Japan showed that less work-related sitting time was associated with higher levels of work efficiency and engagement [6]. By contrast, some studies have shown that sitting time at work was neither associated with lower work productivity [7] nor with presenteeism [7–9]. However, these cross-sectional studies did not consider the time-use composition of these behaviours. As time spent in the workplace setting is limited, examining the associations between reduced occupational sitting time and work productivity indicators without considering the relative effects of the specific activities with which it is replaced may underestimate the benefits of reducing sitting at the workplace. Several experimental studies have shown that sit–stand workstations allowing office workers to switch between sitting and upright postures (i.e. standing or stepping) at work were followed by a decrease in the frequency of work errors [10], an increase in the vitality of work engagement and perceived work performance [11], and improved productivity outcomes [12].

In this context, isotemporal substitution analysis can be informative, as it allows for investigating the outcomes associated with these different activity behaviours within a time-use composition [13]. The analytic methods have been largely applied to understand the potential effects of reallocating sitting time to physical activity on health outcomes [14,15], but only two studies on work performance. A recent study of company workers found that they had a higher work efficiency when replacing workday sedentary behaviours with light-intensity physical activities [16]. By contrast, a study of blue-collar workers showed that reallocating work time from sedentary or light activities to moderate-to-vigorous-intensity physical activity was associated with a higher risk of long-term sickness absence [17]. The difference in the findings aligns with previous research on the ‘physical activity paradox’ [18], suggesting there may be an optimal balance of activities during the working day. For desk workers, re-distributing their prolonged sitting time to other non-sedentary behaviour modes, particularly upright postures (standing/stepping) which can be feasible to achieve through small changes accumulated throughout the workday, may have the potential for enhancing performance at workplaces.

This study estimated changes in desk workers’ presenteeism, absenteeism and engagement when occupational sitting time is reallocated to standing/stepping.

## Methods

This study was approved by the Institutional Ethics Committee of Waseda University (2020-135).

The study participants were recruited online via a Japanese internet research service company (MyVoice Communication, Inc. Tokyo, Japan) in February 2019. This company holds around 1 million individuals who registered as panel members and provided detailed sociodemographic information that is requested to be renewed every half year. The study design and methodology have been previously described elsewhere [9,19]. In brief, individuals aged 20–59 years with full-time jobs (*n* = 45 659) were considered potential participants and randomly selected from the database. To minimize the selection bias due to over-representation of specific demographic subgroups, potential participants were equally stratified by gender and age groups (10-year age band). Those selected participants received an invitation with an introduction to this study topic, aims and process, and a specific link to access the online survey. A total of 3200 completed the survey questionnaire (response rate = 7%) after signing the informed consent form. Each survey participant received reward points valued at 140 yen as an incentive. After excluding those not reported being involved in desk-based employment (*n* = 935) and missing study variables (*n* = 37), 2228 desk workers were included in the analyses.

Two items from the Worker’s Living Activity-Time Questionnaire (WLAQ) [20] were used to assess the relative time (i.e. proportion) spent (i) sitting and (ii) standing/stepping during work hours (excluding commuting time), respectively, in a typical day. The sum of the proportions of sitting and standing/stepping behaviours during work hours is expected to be 100%. The participants were also asked to report the number of days and the average time per day (hours and minutes) worked in the last week. Total time spent in occupational sitting and standing/stepping time per day was computed by multiplying their proportions, respectively, by average work time. WLAQ has been shown strong test–retest reliability with a 1-week interval (intraclass correlation coefficient = 0.87) and moderate validity (Spearman’s *ρ* = 0.67 for sitting and *ρ* = 0.61 for standing/stepping) compared with that measured by activPAL [20].

The work performance was indicated by presenteeism, absenteeism and engagement. The levels of absolute and relative presenteeism and absenteeism were assessed using the Japanese version of the World Health Organization Health and Work Performance Questionnaire (HPQ)—Short Form (https://www.hcp.med.harvard.edu/hpq/info.php). The HPQ can be used to determine the overall work performance in an entire workforce and across different sectors and has been previously validated [21]. Calibration studies have shown the consistency of the HPQ presenteeism metric with archival measures based on supervisor/peer ratings and work audits [21]. Participants were asked to rate their performance during work time on a 0–10 scale, as the measure of absolute presenteeism. The score of absolute presenteeism was rescaled from 0 (presence with the lowest productivity) to 100 (presence with the highest productivity) based on the scoring procedure. Relative presenteeism was measured as a ratio of a worker’s actual performance to the average performance of most workers at the same occupation (as perceived by the participant), with higher scores indicating lower relative presenteeism (i.e. better relative productivity). The range of relative presenteeism was restricted between 0.25 for the lowest relative performance (i.e. 25% or less of most workers’ performance) and 2.00 for the highest relative performance (i.e. 200% or more of most workers’ performance).

Self-reported absenteeism using the HPQ has previously shown moderate-to-strong correlations (*r* = 0.66 and 0.71 for railroad engineers and reservation agents, respectively) with the absenteeism identified by supervisors’ or employers’ payroll records [21]. Absolute absenteeism in the HPQ was assessed as the difference between the number of hours workers worked in the previous 4 weeks and that expected by their supervisors or employers. A negative value in absolute absenteeism indicated an individual working more than expected, while a positive value indicated an individual working less than expected. Relative absenteeism was assessed as absolute absenteeism divided by their supervisors’ expected work hours (i.e. a proportion of expected hours), and ranged from a negative value (an individual works more than expected) to one (always absent). Details on presenteeism and absenteeism were shown in the scoring guideline (https://www.hcp.med.harvard.edu/hpq/info.php).

Work engagement was measured using the Japanese version of the Utrecht Work Engagement Scale (UWES), which is widely used in previous research. The Japanese version of the UWES has been previously found to have high internal consistency (*α* = 0.92), moderate test–retest reliability (*r* = 0.66) with a 2-month recall period, and acceptable construct validity while compared with job satisfaction, strain and burnout assessed using other validated questionnaires [22]. The UWES consisted of 17 items, including three dimensions—vigour (6 items), dedication (5 items) and absorption (6 items). Vigour is characterized by high levels of energy and mental resilience while working. Dedication indicates being firmly involved in one’s work and experiencing a sense of significance and pride. Absorption refers to being fully concentrated and happily engrossed in one’s work. All the items in the questionnaire were scored on a 7-point Likert scale ranging from 0 (‘never’) to 6 (‘always’). Higher scores for overall and each dimension indicate a higher level of work engagement.

Sociodemographic characteristics such as gender, age group (using 10-year bands), marital status (not married or married), educational attainment (below tertiary education or tertiary education) and individual annual income (<4 million or ≥4 million yen) were included as covariates. The number of workers in the workplace (<29, 30–99, ≥100 workers or missing) was also considered, as different scales of the workplace (small, medium and large) may offer differential opportunities for interaction and knowledge exchange that may impact work performance.

Multivariable linear regression analyses, including partition and isotemporal substitution models, were used to examine the associations of occupational sitting and standing/stepping time with work presenteeism, absenteeism and engagement, after checking the normally assumption [23]. The partition models simultaneously included both occupational sitting and standing/stepping behaviours in the model without adjusting for total work hours (i.e. no upper limit for work hours). The coefficient for each behaviour represents the effects of adding, rather than reallocating, the time for one behaviour. In other words, the coefficient for occupational sitting time refers to the effect of increasing this type of behaviour while holding standing/stepping time constant. The isotemporal model aimed to estimate the effect of reallocating the behaviours by removing the specific replaced behaviour and keeping the work hours constant in the model [24]. The isotemporal substitution models examined the effect of reallocating 1 hour as a unit of occupational sitting to standing/stepping. In the current study, the results of the single-activity model (i.e. the model includes one specific behaviour each time) cannot be demonstrated uniquely, as its activity components in the regression models would be the same as that in the isotemporal substitution models in the case of including only two types of behaviours. A similar approach has been shown in a previous study [25]. The unstandardized regression coefficients (*B*) and their 95% confidence intervals (CIs) were estimated. IBM SPSS Statistics 20.0 (IBM Corp, Armonk, NY) was used to conduct the analyses, and the significance level was set at *P* < 0.05.

## Results

Table 1 presents the characteristics of the 2228 study participants (mainly working as company employees). The mean work time per day was 8.3 (±1.9) hours, of which most of the time was spent sitting. On average, the participants reported a relatively high absolute presenteeism score, equal performance compared with co-workers, longer work hours than their supervisors/employers expected and relatively low work engagement.

**Table 1.** Characteristics of the study participants (*n* = 2228)

	*n* (%)
Gender	
Men	1080 (48)
Women	1148 (52)
Age (years)	
20–29	523 (23)
30–39	569 (26)
40–49	570 (26)
50–59	566 (25)
Marital status	
Not married	1239 (56)
Married	989 (44)
Educational attainment	
Below tertiary education	310 (14)
Tertiary education	1918 (86)
Individual annual income	
<4 million yen	1143 (51)
≥4 million yen	1085 (49)
Workplace scale	
Small (≤29 workers)	529 (24)
Medium (30–99 workers)	319 (14)
Large (≥100 workers)	1308 (59)
Missing	72 (3)
	Mean (SD)
Work time (hour/day)	8.3 (1.9)
Occupational sitting (hour/day)	6.6 (1.9)
Occupational standing or stepping (hour/day)	1.7 (1.3)
Work performance	
Presenteeism score	
Absolute (0–100)	57.8 (17.1)
Relative (0.25–2.00)	1.0 (0.2)
Absenteeism score	
Absolute	−6.0 (59.1)
Relative	−0.4 (1.8)
Engagement scale	
Overall (0–6)	2.7 (1.1)
Vigour (0–6)	2.6 (1.1)
Dedication (0–6)	2.8 (1.2)
Absorption (0–6)	2.7 (1.1)

SD, standard deviation.

The partition models for the associations of occupational sitting and standing/stepping behaviours with work performance are shown in Table 2. Occupational sitting was significantly associated with a lower absolute presenteeism score (i.e. less productivity) after adjusting for covariates and occupational standing/stepping. Hours spent in occupational sitting and standing/stepping behaviours were negatively associated with absolute absenteeism, indicating an individual worked more hours than expected. An increase in occupational sitting hours was associated with a decrease in work engagement, including overall and in most dimensions—vigour and dedication; by contrast, an increase in occupational standing/stepping behaviour was associated with an increase in overall and dimension-specific work engagement.

**Table 2.** Partition models examining the associations of occupational sitting and standing/stepping hours with work presenteeism, absenteeism and engagement

Work performance	Occupational sitting			Occupational standing/stepping		
	*B*	(95% CI)	*P*	*B*	(95% CI)	*P*
Presenteeism score						
Absolute	**−0.458**	**(−0.862, −0.053)**	**0.027**	-0.161	(**−**0.752, 0.430)	0.59
Relative	0.001	(**−**0.004, 0.007)	0.63	0.004	(-0.005, 0.012)	0.39
Absenteeism score						
Absolute	**−4.185**	**(−5.581, −2.789)**	**<0.001**	**−2.445**	**(−4.484, −0.405)**	**0.019**
Relative	0.027	(**−**0.017, 0.071)	0.22	0.019	(**−**0.045, 0.084)	0.55
Engagement scale						
Overall	**−0.030**	**(−0.055, −0.005)**	**0.020**	**0.057**	**(0.020, 0.093)**	**0.002**
Vigour	**−0.035**	**(−0.060, −0.010)**	**0.006**	**0.056**	**(0.019, 0.093)**	**0.003**
Dedication	**−0.042**	**(−0.071, −0.014)**	**0.004**	**0.050**	**(0.008, 0.091)**	**0.019**
Absorption	**−**0.014	(**−**0.039, 0.012)	0.28	**0.064**	**(0.026, 0.101)**	**0.001**

*B*, unstandardized coefficient; CI, confidence interval.

All the models were adjusted for gender, age group, marital status, educational attainment, individual annual income, and workplace scale; 95% CIs that do not include zero are highlighted in bold.

The isotemporal substitution models show that each hour of occupational sitting reallocated to standing/stepping was favourably associated with overall work engagement and by dimension—vigour, dedication and absorption (Table 3). No significant associations were found for presenteeism or absenteeism.

**Table 3.** Isotemporal substitution models examining the associations of replacing occupational sitting with standing/stepping hours on work presenteeism, absenteeism and engagement

Work performance	*B*	(95% CI)	*P*
Presenteeism score			
Absolute	0.297	(−0.276, 0.869)	0.31
Relative	0.002	(−0.006, 0.010)	0.58
Absenteeism score			
Absolute	1.740	(−0.233, 3.713)	0.084
Relative	−0.008	(−0.070, 0.054)	0.81
Engagement scale			
Overall	**0.087**	**(0.051, 0.122)**	**<0.001**
Vigour	**0.092**	**(0.056, 0.127)**	**<0.001**
Dedication	**0.092**	**(0.052, 0.132)**	**<0.001**
Absorption	**0.078**	**(0.042, 0.114)**	**<0.001**

*B*, unstandardized coefficient; CI, confidence interval.

All the models were adjusted for gender, age group, marital status, educational attainment, individual annual income, workplace scale and work hours; 95% CIs that do not include zero are highlighted in bold.

## Discussion

Examining the associations of occupational sitting and standing/stepping behaviours with work performance using the partition and isotemporal substitution approach, we found that, using partition models, more time spent in occupational sitting was associated with a lower absolute presenteeism score (i.e. less productivity), but not its relative measure. This is consistent with the findings of an earlier study from Japan showing that workers who engaged in a longer work-related sitting time had higher presenteeism [6]. The more marked associations found for the absolute presenteeism score than the relative one may be attributable to the larger variance in the absolute than the relative measure. The difference in outcomes for the absolute and relative measures aligns with the argument that a relative presenteeism score may be more useful when it is applied to heterogeneous populations such as workers with different work hours demanded [26] and those with shift work [27]. However, one study from Australia showed longer occupational sitting time was associated with lower presenteeism [28]. The difference in results between this study and the Australian study may be attributable to the large proportion of non-full-time workers (~40%) in the Australian study, whose occupational behavioural patterns and work performance evaluation may differ from full-time workers. Some previous studies found no associations of occupational sitting time with either presenteeism [7,9] or lost work productivity [7]. In contrast to the findings of experimental studies which found that installation of a sit–stand workstation reduced workers’ sitting time and increased their work performance [10,11], we found no association with presenteeism when occupational sitting was reallocated to standing/stepping. Applying different research designs and using different questionnaires for assessing sitting behaviours and work performance [29] may account partially for these inconsistent findings.

In the partition models that held time spent in the other behaviour constant, we also found that more time spent in occupational sitting and standing/stepping was associated with lower absolute absenteeism, indicating higher-than-expected work hours. Given that there was no maximum of total work hours in partition models, the finding that higher-than-expected work hours were associated with both occupational sitting and standing/stepping may simply reflect the longer work hours. It has also been argued that such an adding effect of the partition models may be confounded by the total time for behaviours [24]. The associations with absolute absenteeism were not observed for occupational sitting or standing/stepping in the isotemporal substitution models, which control for total work hours, supporting this potential explanation. The findings across the two models highlight the importance of considering the time-use composition structure of workplace sitting, standing and stepping.

The partition models indicated that occupational sitting (negatively) and standing/stepping (positively) were associated with work engagement. This aligns with previous findings showing that less time spent in prolonged occupational sitting was associated with a higher level of work engagement [5,6]. When reallocating occupational sitting to standing/stepping, positive associations were also found for work engagement, both overall and in each dimension. Our findings were consistent with evidence from a trial of sit–stand workstations, which showed an increase in the vigour dimension of work engagement when workers were able to increase their workplace standing time [11]. Meta-analyses have reported that workers with more time in the workplace sitting had 23–47% higher risks of low back pain [30,31] and a 73% higher risk of neck/shoulder pain [31]. A lower risk of physical pains in desk workers who involved in less sedentary time may have higher levels of energy and work concentration [32]. Furthermore, time spent standing and stepping may increase workplace social interaction [33], social capital and resources of work engagement, contributing to an enhancement of involvement in work [34]. The combination of lower level of physical pain and higher level of social interaction may amplify the benefits of reallocating sitting time to standing/stepping among desk workers in countries/areas where a limited population working from home. These findings suggest that creating and offering opportunities to support workers to engage in more time standing and stepping at work by designing open-plan offices [35] and providing behavioural cues, for example, e-newsletters and managerial support [36] may be beneficial for work engagement without detrimentally affecting presenteeism or absenteeism [37].

This study is the first to investigate the associations of reallocating occupational sitting time to stepping/standing with desk workers’ performance using isotemporal substitution models. There are some limitations to this study. We were unable to infer a cause-and-effect relationship between sitting time, standing/stepping and work performance due to the cross-sectional design. The study participants were recruited from the internet, with a low response rate, which may lead to limited generalizability due to self-selection [38]. Replications with a nationally representative sample can corroborate the findings. There were some residual confounding factors, for example, physical disorders need to be considered when investigating the relationships between behaviours and work performance. Self-reported measures of behaviours and work performance indicators may be subject to bias due to erroneous recall and/or social desirability. Research using objective measures for behaviours and work-related performance (e.g. monitors and work-related reports) is required. The design of question did not allow for separating occupational standing and stepping behaviours; it was also not able to identify the types of activities that workers were doing while sitting. Previous research suggested that standing and stepping may have different effects on health risks [39] and work performance. A study that measured sitting time using accelerometers and logbooks also reported marked differences in the associations of work presenteeism with sitting time during work hours for doing work and having lunch [8]. Future studies are encouraged to apply measures of sedentary and upright behaviours, with information on the time of day and type of work activity, to provide a better understanding of the relationships between sitting-to-standing or -stepping reallocation and work performance indicators.

In conclusion, there are putative benefits of reallocating occupational sitting to standing/stepping on work engagement, with limited impact on presenteeism or absenteeism. Work practices that support more time being spent standing and stepping during work hours, with decreased sitting behaviours, are likely to be helpful in improving desk workers’ overall performance. Longitudinal studies using both self-reported and objective measures of these physical behaviours and indicators of work performance are required to corroborate the relationships identified, to inform the development of strategies that can benefit desk workers and their productivity.
